# Supplementary material for: R-Score: A New Parameter to Assess the Quality of Variants’ Calls Assessed by NGS Using Liquid Biopsies
Source: Biology (Basel). 2021 Sep 24;10(10):954. doi: 10.3390/biology10100954 (PMC8533561; doi:10.3390/biology10100954)
Supplement: Supplementary file 1 [file biology-10-00954-s001.zip › biology-1339819-supplementary.pdf]

biology

MDPI

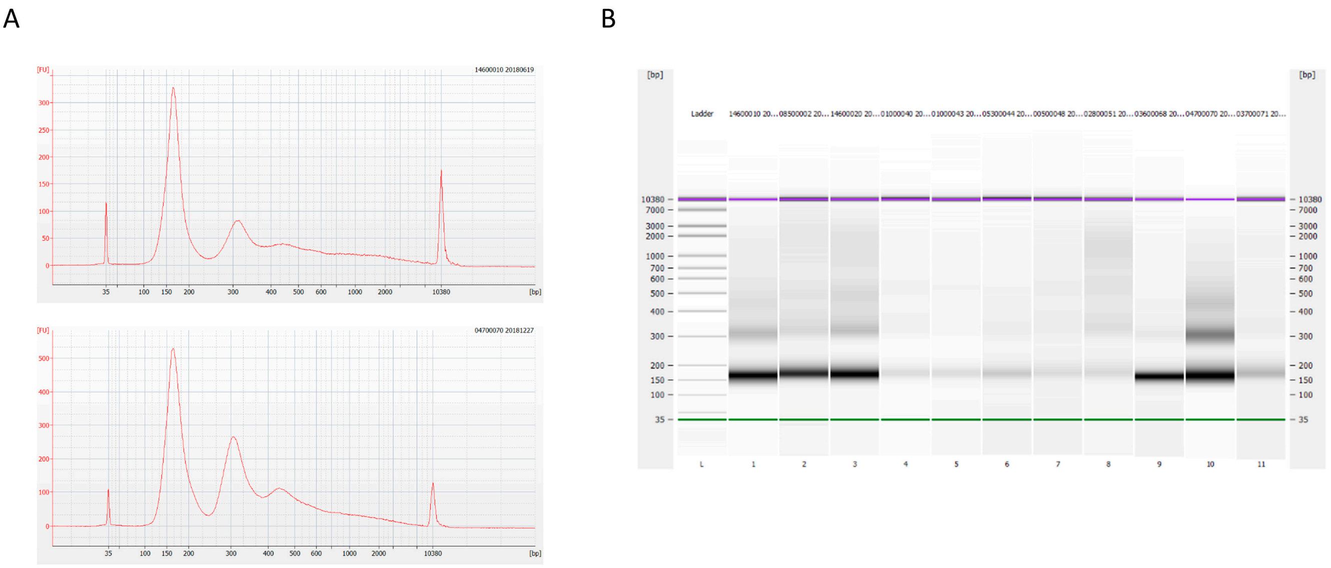

Figure S1. (**A**) Two samples electropherogram showing a 180 bp peak corresponding to low fragment cfDNA and peaks corresponding to mono-, di-, and tri-nucleosomes. (**B**) Gel-like image of cfDNA samples analysed with Bioanalyzer 2100.

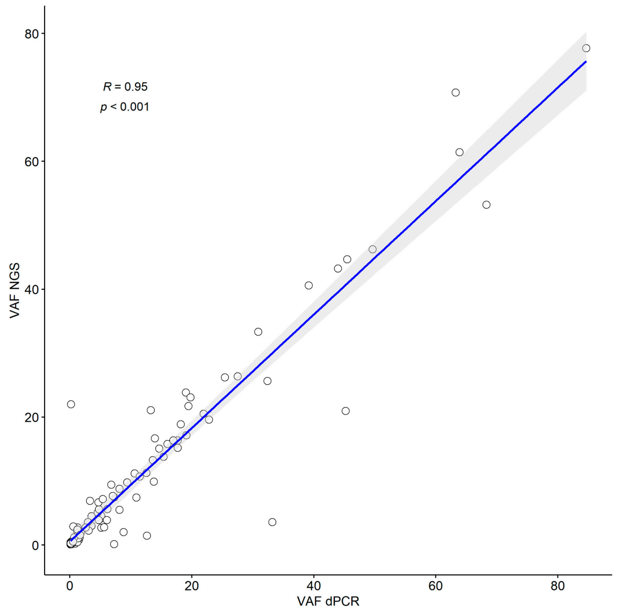

Figure S2. Correlation between variant allele fraction assessed by dPCR and Oncomine-NGS. Linear regression line is shown in black and the 95% confidence interval is shaded in grey. Pearson's correlation coefficient and p-value are shown in the graph.

Table S1. Effect size and p-values for VAF, MAPD, and R-score parameters when assessing significant differences between concordant and discordant calls.

	**Effect Size**	***P*-value**
VAF value	0.28	0.001
MAPD value	–0.48	< 0.001
R-score	0.57	< 0.001

Statistics for the Mann–Whitney U test

Table S2. Table with all *EGFR* mutations detected by dPCR, NGS-Oncomine, and NGS-GeneReader with VAF values for each assay and cfDNA concentration.

Patient	cfDNA (ng/µL)	EGFR initial mutation	dPCR	VAF_dPCR	NGS-Oncomine	VAF_NGS-Oncomine	MAPD_NGS-Oncomine	NGS-GeneReader	VAF_NGS_GeneReader	R-score
1	1.060	c.2236_2250del15	c.2236_2250del15	7.007	wt	0	4.436	wt	0	
			c.2369C>T	1.400	wt	0	4.436	wt	0	
2	0.038	c.2573T>G	c.2573T>G	1.500	c.2573T>G	0.790	0.281			−0.449
3	0.102	c.2240_2257del18	c.2240_2257del18	43.943	c.2240_2257del18	43.210	0.208			−2.318
			c.2369C>T	0.100	wt	0	0.208			
4	0.108	c.2235_2249del15	c.2236_2250del15	19.096	c.2236_2250del15	17.143	0.204			−1.924
			c.2369C>T	8.798	c.2369C>T	2.031	0.204			−0.998
5	1.090	c.2235_2249del15	c.2235_2249del15	0.216	wt	0	0.167	wt	0	
6	NA	c.2236_2250del15	c.2236_2250del15	0.838	wt	0	0.190			
7	0.006	c.2235_2249del15	c.2235_2249del15	3.147	wt	0	0.287			
8	0.127	c.2235_2249del15	c.2236_2250del15	4.965	c.2236_2250del15	4.078	0.174			−1.370
			c.2369C>T	0.100	wt	0	0.174			
9	0.751	c.2573T>G	c.2573T>G	7.245	c.2573T>G	0.113	0.525			0.667
10	0.472	c.2235_2249del15	c.2235_2249del15	10.895	c.2235_2249del15	7.430	0.244			−1.484
			c.2369C>T	1.603	c.2369C>T	1.210	0.244			−0.695
11	0.284	c.2582T>A	c.2582T>A	1.181	c.2582T>A	2.150	0.261			−0.916
12	0.210	c.2235_2249del15	c.2235_2249del15	25.429	c.2235_2249del15	26.222	0.243			−2.033
			c.2369C>T	3.554	c.2369C>T	4.494	0.243			−1.267
13	1.800	c.2235_2249del15/c.2303G>T	c.2369C>T	0.396	wt	0	0.930	wt	0	
14	1.120	c.2235_2249del15	c.2235_2249del15	1.260	c.2235_2249del15	1.308	0.169			−0.889
			c.2369C>T	0.200	c.2369C>T	0.475	0.169			−0.449

15	0.320	c.2235_2249del15	c.2235_2249del15	9.327	wt	0	1.897	wt	0	
16	1.390	c.2240_2254del15	c.2240_2254del15	12.620	c.2240_2254del15	1.450	0.244	wt	0	−0.774
			c.2369C>T	0.503	c.2369C>T	0.240	0.244	c.2369C>T	0.230	0.007
17	0.328	c.2240_2257del18	c.2240_2257del18	19.015	c.2240_2257del18	23.850	0.189			−2.101
			c.2369C>T	0.589	wt	0	0.189			
18	0.431	c.2236_2250del15	c.2236_2250del15	45.464	c.2236_2250del15	44.690	0.367			−2.086
			c.2369C>T	18.195	c.2369C>T	18.860	0.367			−1.711
19	0.480	c.2236_2250del15	c.2236_2250del15	84.664	c.2236_2250del15	77.668	0.334			−2.366
			c.2369C>T	17.667	c.2369C>T	15.180	0.334			−1.658
20	7.380	c.2582T>A	c.2582T>A	0.100	c.2582T>A	0.105	0.306			0.464
21	0.799	c.2240_2257del18	c.2240_2257del18	0.681	c.2240_2257del18	0.760	0.212			−0.554
			c.2369C>T	0.121	c.2369C>T	0.400	0.212			−0.276
22	1.320	c.2156G>C	c.2156G>C	3.546	c.2156G>C	2.970	0.229			−1.113
23	0.717	c.2156G>C	c.2156G>C	13.752	c.2156G>C	9.910	0.328			−1.480
24	0.805	c.2236_2250del15	c.2236_2250del15	15.377	c.2236_2250del15	13.790	0.185			−1.872
			c.2369C>T	6.792	c.2369C>T	9.440	0.185			−1.708
25	0.038	c.2573T>G	c.2573T>G	1.873	wt	0	0.408	wt	0	
26	0.326	c.2310_2311insGGT	c.2310_2311insGGT	0.293	wt	0	0.388	wt	0	
27	0.170	c.2235_2249del15	c.2235_2249del15	49.641	c.2235_2249del15	46.225	0.229			−2.305
			c.2369C>T	3.321	c.2369C>T	6.915	0.229			−1.480
28	0.580	c.2237_2255>T	c.2237_2255>T	5.152	c.2237_2255>T	4.570	0.295			−1.190
29	1.200	c.2573T>G	c.2573T>G	68.296	c.2573T>G	53.190	0.587			−1.957
30	0.990	c.2235_2249del15	c.2235_2249del15	5.173	c.2235_2249del15	2.680	0.471			−0.755
			c.2369C>T	0.100	wt	0	0.471			
31	0.182	c.2236_2250del15	c.2236_2250del15	13.938	c.2236_2250del15	16.670	0.354			−1.673
32	0.610	c.2239_2256del18	c.2239_2256del18	0.579	c.2236_2250del15	0.820	0.422			−0.288
33	1.030	c.2573T>G	c.2573T>G	13.615	c.2573T>G	13.250	0.375			−1.548

34	4.980	c.2235_2249del15	c.2235_2249del15	63.279	c.2235_2249del15	70.730	1.797			−1.595
			c.2369C>T	0.100	wt	0	1.797			
35	3.450	c.2239_2248TTAAGAG AAG>C	c.2239_2248TTAAGAG AAG>C	0.883	c.2239_2248TTAAGAG AAG>C	0.210	0.168	wt	0	−0.097
36	0.100	c.2235_2249del15	c.2235_2249del15	7.287	c.2235_2249del15	7.460	0.456			−1.214
37	0.952	c.2573T>G	c.2573T>G	32.414	c.2573T>G	25.630	0.429			−1.776
38	0.742	c.2240_2257del18	c.2240_2257del18	0.681	c.2240_2257del18	0.485	0.283			−0.234
			c.2369C>T	0.121	c.2369C>T	0.295	0.283			−0.017
39	0.473	c.2235_2249del15	c.2235_2249del15	5.626	c.2235_2249del15	2.778	0.308			−0.955
			c.2369C>T	0.509	wt	0	0.308			
40	0.981	c.2573T>G	c.2573T>G	8.140	c.2573T>G	5.479	0.265			−1.315
			c.2369C>T	3.102	c.2369C>T	2.270	0.265			−0.933
41	1.840	c.2573T>G/c.2303G>T	c.2573T>G	17.674	c.2573T>G	16.337	0.278			−1.769
			c.2303G>T	13.270	c.2303G>T	21.070	0.278			−1.880
			c.2369C>T	4.578	c.2369C>T	5.050	0.278			−1.259
42	0.688	c.2235_2249del15	c.2236_2250del15	0.365	c.2236_2250del15	0.321	0.221			−0.161
43	0.232	c.2235_2249del15	c.2235_2249del15	1.703	wt	0	0.284			
44	1.110	c.2573T>G	c.2573T>G	0.510	wt	0	1.405	wt	0	
			c.2369C>T	0.519	wt	0	1.405	wt	0	
45	0.639	c.2236_2250del15	c.2236_2250del15	45.218	c.2236_2250del15	20.950	0.420	c.2236_2250del15	20.300	−1.698
			c.2369C>T	27.516	c.2369C>T	26.380	0.420	c.2369C>T	27.000	−1.798
46	0.518	c.2239_2256del18	c.2239_2256del18	19.800	c.2236_2252delGAATT AAGAGAAGCAACinsAT (COSM26680)	23.080	0.098	c.2236_2252delGAATT AAGAGAAGCAACinsAT (COSM26680)	14.430	−2.372
			c.2369C>T	15.993	c.2369C>T	15.810	0.098	c.2369C>T	12.900	−2.208
47	0.280	c.2573T>G	c.2573T>G	63.908	c.2573T>G	61.400	0.145	c.2573T>G	57.400	−2.627
			c.2369C>T	22.795	c.2369C>T	19.610	0.145	c.2369C>T	18.500	−2.131

48	0.735	c.2239_2256del18	c.2239_2256del18	0.688	wt	0	0.279			
			c.2369C>T	0.326	wt	0	0.279			
49	7.950	c.2573T>G	c.2573T>G	19.459	c.2573T>G	21.700	0.171	c.2573T>G	18.000	−2.103
50	0.244	c.2573T>G	c.2573T>G	4.337	wt	0	0.424			
51	2.380	c.2573T>G	c.2573T>G	9.410	c.2573T>G	9.800	0.096	c.2573T>G	8.560	−2.009
			c.2369C>T	1.277	c.2369C>T	1.350	0.096	c.2369C>T	0.910	−1.148
52	0.080	c.2236_2250del15	c.2236_2250del15	1.300	c.2236_2250del15	2.730	0.127	c.2236_2250del15	0.400	−1.332
			c.2369C>T	0.360	wt	0	0.127	c.2369C>T	1.600	
53	0.340	c.2573T>G	c.2573T>G	0.211	c.2573T>G	0.400	0.108	c.2573T>G	0.420	−0.569
			c.2369C>T	0.174	wt	0	0.108	wt	0	
54	0.210	c.2573T>G	c.2573T>G	10.607	c.2573T>G	11.200	0.116	c.2573T>G	9.790	−1.985
			c.2369C>T	4.777	c.2369C>T	5.580	0.116	c.2369C>T	2.630	−1.682
55	0.190	c.2236_2250del15	c.2236_2250del15	33.190	c.2236_2250del15	3.570	0.740	c.2236_2250del15	26.860	−0.683
			c.2369C>T	13.602	wt	0	0.740	c.2369C>T	11.200	
56	2.240	c.2239_2253del15	c.2239_2253del15	4.709	c.2239_2253del15TTA<br>AGAGAAGCAACA	6.660	0.729	c.2239_2253del15TTA<br>AGAGAAGCAACA	3.780	−0.961
			c.2369C>T	6.061	c.2369C>T	3.880	0.729	c.2369C>T	4.660	−0.726
57	0.891	c.2235_2249del15	c.2369C>T	0.302	wt	0	0.639	wt	0	
58	0.475	c.2582T>A/c.2573T>G	c.2582T>A	1.078	c.2582T>A	1.020	0.330	c.2582T>A	1.230	−0.490
			c.2573T>G	0.410	wt	0	0.330	c.2573T>G	0.540	
			c.2369C>T	0.175	wt	0	0.330	wt	0	
59	1.040	c.2236_2253del18	c.2236_2253del18	0.192	L747S	21.980	0.358	L747S	28.000	−1.788
			c.2369C>T	12.537	c.2369C>T	11.260	0.358	c.2369C>T	11.000	−1.498
60	2.400	c.2573T>G	c.2573T>G	18.707	wt	0	0.355			
			c.2369C>T	0.166	wt	0	0.355			
61	0.360	c.2573T>G	c.2573T>G	2.962	c.2573T>G	3.570	0.471	c.2573T>G	5.340	−0.880
62	1.760	c.2573T>G	c.2573T>G	0.198	c.2573T>G	0.300	0.230	wt	0	−0.115

			c.2369C>T	0.430	wt	0	0.230	wt	0	
63	1.930	c.2236_2250del15	c.2236_2250del15	11.453	c.2236_2250del15	10.700	0.244	c.2236_2250del15	6.050	−1.642
			c.2369C>T	5.409	c.2369C>T	7.170	0.244	c.2369C>T	3.700	−1.468
64	1.050	c.2573T>G	c.2573T>G	8.119	c.2573T>G	8.800	0.234	c.2573T>G	7.100	−1.575
			c.2369C>T	4.583	wt	0	0.234	c.2573T>G	3.400	
65	1.110	c.2573T>G	c.2573T>G	0.570	wt	0	0.527	wt	0	
			c.2369C>T	0.614	c.2369C>T	0.480	0.527	wt	0	0.041
66	1.330	c.2573T>G	c.2573T>G	0.553	wt	0	0.770			
67	1.480	c.2240_2257del18	c.2240_2257del18	30.900	c.2240_2257del18	33.330	0.647	c.2240_2257del18	27.400	−1.712
			c.2369C>T	1.605	wt	0	0.647	c.2369C>T	1.830	
68	1.350	c.2573T>G	c.2573T>G	0.149	wt	0	0.455	wt	0	
69	1.170	c.2238_2248>GC	c.2238_2248>GC	1.800	c.2238_2248>GC	1.980	0.517	wt	0	−0.583
70	3.000	c.2235_2249del15	c.2235_2249del15	14.660	c.2235_2249del15	15.050	0.426			−1.548
71	0.429	c.2573T>G	c.2573T>G	1.066	c.2573T>G	0.450	0.289	c.2573T>G	1.350	−0.192
			c.2369C>T	1.231	c.2369C>T	0.430	0.289	c.2369C>T	1.000	−0.173
72	1.620	c.2236_2250del15	c.2236_2250del15	39.170	c.2236_2250del15	40.600	0.308	c.2236_2250del15	36.920	−2.120
73	0.638	c.2573T>G	c.2573T>G	0.175	c.2573T>G	0.100	0.246	wt	0	0.391
			c.2369C>T	0.185	wt	0	0.246	wt	0	
74	0.442	c.2573T>G	c.2573T>G	0.586	c.2543C>T	2.900	2.032			−0.154
			c.2369C>T	0.547	wt	0	2.032			
75	0.650	c.2235_2249del15	c.2235_2249del15	1.420	c.2235_2249del15	1.890	0.498	c.2235_2249del15	1.680	−0.579
			c.2369C>T	0.320	wt	0	0.498	wt	0	
76	0.215	c.2573T>G	c.2573T>G	0.712	wt	0	0.205			
77	0.100	c.2235_2249del15	c.2235_2249del15	4.720	c.2235_2249del15	3.900	0.459	c.2235_2249del15	2.440	−0.929
78	0.531	c.2573T>G	c.2573T>G	0.135	c.2573T>G	0.150	0.167	wt	0	0.047
			c.2369C>T	0.203	wt	0	0.167	wt	0	
79	0.226	c.2235_2249del15	c.2235_2249del15	0.838	wt	0	0.282			

			c.2369C>T	0.427	wt	0	0.210			
80	1.400	c.2573T>G	c.2573T>G	0.157	c.2573T>G	0.240	0.197			−0.086
81	0.875	c.2573T>G	c.2573T>G	16.971	c.2573T>G	16.360	0.387	c.2573T>G	14.200	−1.626
			c.2369C>T	7.038	c.2369C>T	7.660	0.387	c.2369C>T	3.750	−1.297
82	12.200	c.2573T>G	c.2573T>G	21.911	c.2573T>G	20.530	0.339	c.2573T>G	21.910	−1.782
			c.2369C>T	2.591	c.2369C>T	2.720	0.339	c.2369C>T	2.620	−0.904
83	1.040	c.2235_2249del15	c.2235_2249del15	1.239	c.2235_2249del15	2.400	0.191	c.2235_2249del15	1.450	−1.099
			c.2369C>T	1.380	c.2369C>T	1.035	0.191	c.2369C>T	0.650	−0.734
84	1.250	c.2237_2255>T	c.2237_2255>T	0.711	c.2237_2255>T	1.158	0.160	wt	0	−0.860
			c.2369C>T	0.532	c.2369C>T	0.540	0.160	wt	0	−0.528
85	4.690	c.2235_2249del15	c.2235_2249del15	6.175	c.2235_2249del15	5.630	0.254			−1.346
			c.2369C>T	1.662	c.2369C>T	1.57	0.254			−0.790
